# T cell responses to repeated SARS-CoV-2 vaccination and breakthrough infections in patients on TNF inhibitor treatment: a prospective cohort study

**DOI:** 10.1016/j.ebiom.2024.105317

**Published:** 2024-09-10

**Authors:** Asia-Sophia Wolf, Kristin H. Bjørlykke, Hilde S. Ørbo, Sabin Bhandari, Guri Solum, Ingrid Fadum Kjønstad, Ingrid Jyssum, Unni C. Nygaard, Anja Bråthen Kristoffersen, Ingrid E. Christensen, Sarah E. Josefsson, Katrine Persgård Lund, Adity Chopra, Julie Røkke Osen, Viktoriia Chaban, Anne T. Tveter, Joseph Sexton, Tore K. Kvien, Jørgen Jahnsen, Espen A. Haavardsholm, Gunnveig Grødeland, John Torgils Vaage, Sella A. Provan, Hassen Kared, Fridtjof Lund-Johansen, Ludvig A. Munthe, Silje Watterdal Syversen, Guro Løvik Goll, Kristin Kaasen Jørgensen, Siri Mjaaland

**Affiliations:** a Division of Infection Control, Section for Immunology, Norwegian Institute of Public Health, Oslo, Norway; b Department of Gastroenterology, Akershus University Hospital, Lørenskog, Norway; c Institute of Clinical Medicine, University of Oslo, Oslo, Norway; d Center for Treatment of Rheumatic and Musculoskeletal Diseases (REMEDY), Diakonhjemmet Hospital, Oslo, Norway; e Division of Infection Control, Section for Modelling and Bioinformatics, Norwegian Institute of Public Health, Oslo, Norway; f Department of Immunology, Oslo University Hospital, Oslo, Norway; g KG Jebsen Centre for B Cell Malignancy, University of Oslo, Oslo, Norway; h Faculty of Health Sciences, Oslo Metropolitan University, Oslo, Norway; i Section for Public Health, Inland Norway University of Applied Sciences, Norway; j Institute of Health and Society, University of Oslo, Norway

**Keywords:** TNF inhibitors, SARS-CoV-2, Vaccination, T cells, Inflammatory bowel disease, Arthritis

## Abstract

**Background:**

Understanding cellular responses to SARS-CoV-2 immunisations is important for informing vaccine recommendations in patients with inflammatory bowel disease (IBD) and other vulnerable patients on immunosuppressive therapies. This study investigated the magnitude and quality of T cell responses after multiple SARS-CoV-2 vaccine doses and COVID-19 breakthrough infection.

**Methods:**

This prospective, observational study included patients with IBD and arthritis on tumour necrosis factor inhibitors (TNFi) receiving up to four SARS-CoV-2 vaccine doses. T cell responses to SARS-CoV-2 peptides were measured by flow cytometry before and 2–4 weeks after vaccinations and breakthrough infection to assess the frequency and polyfunctionality of responding cells, along with receptor-binding domain (anti-RBD) antibodies.

**Findings:**

Between March 2, 2021, and December 20, 2022, 143 patients (118 IBD, 25 arthritis) and 73 healthy controls were included. In patients with either IBD or arthritis, humoral immunity was attenuated compared to healthy controls (median anti-RBD levels 3391 vs. 6280 BAU/ml, p = 0.008) after three SARS-CoV-2 vaccine doses. Patients with IBD had comparable quantities (median CD4 0.11% vs. 0.11%, p = 0.26, CD8 0.031% vs. 0.047%, p = 0.33) and quality (polyfunctionality score: 0.403 vs. 0.371, p = 0.39; 0.105 vs. 0.101, p = 0.87) of spike-specific T cells to healthy controls. Patients with arthritis had lower frequencies but comparable quality of responding T cells to controls. Breakthrough infection increased spike-specific CD8 T cell quality and T cell responses against non-spike peptides.

**Interpretation:**

Patients with IBD on TNFi have T cell responses comparable to healthy controls despite attenuated humoral responses following three vaccine doses. Repeated vaccination and breakthrough infection increased the quality of T cell responses. Our study adds evidence that, in the absence of other risk factors, this group may in future be able to follow the general recommendations for COVID-19 vaccines.

**Funding:**

10.13039/501100006095South-Eastern Norway Regional Health Authority, 10.13039/100016302Coalition for Epidemic Preparedness Innovations (CEPI), 10.13039/501100021078Norwegian Institute of Public Health, 10.13039/501100012446Akershus University Hospital, Diakonhjemmet Hospital.

**Research in context**

**Evidence before this study**

We searched PubMed and medRxiv for studies published in English between January 1, 2021, and December 22, 2023, using the terms “inflammatory bowel disease”, “arthritis”, “immune-mediated inflammatory disorder”, “tumour necrosis factor alpha”, “SARS-CoV-2”, “COVID-19”, “vaccination”, “cellular immunity”, “T cell responses”, “immunosuppressive therapy”, “immunocompromised”, “breakthrough infection”, and “omicron” to identify studies on T cell immune responses following SARS-CoV-2 vaccination and breakthrough infection in patients treated with TNFi. We identified ten studies and one meta-analysis assessing T cell responses in patients on TNFi therapy following three SARS-CoV-2 vaccine doses. Overall, these studies showed conflicting results, where some suggested that patients on TNFi therapies had reduced T cell activity compared to healthy controls and others found comparable results between TNFi-treated patients and healthy controls or individuals on other immunomodulating therapies. Two additional studies investigated cellular responses after up to four vaccine doses; one showed no impact on the magnitude of spike-specific CD4 responses following a third and fourth dose, the other demonstrated increased CD8 responses with additional doses. The majority of these studies had small sample sizes, and/or did not consider breakthrough infections. Notably, in most prior studies, T cell responses were measured indirectly by interferon gamma assays and did not differentiate between CD4 and CD8 responses or assess the quality of these responses. The magnitude and quality of T cell responses following repeated vaccination with up to four doses and breakthrough infections have not previously been described. T cell polyfunctionality, defined as co-expression of two or more activation markers or cytokines, has been found to be an important measure of protective vaccine responses in multiple diseases including HIV, tuberculosis, and other viral, bacterial, and parasitic infections.

**Added value of this study**

This study demonstrates that although patients with inflammatory bowel disease (IBD) on TNFi have attenuated humoral immune responses after multiple SARS-CoV-2 vaccines, the magnitude and quality of T cell responses are comparable to responses seen in healthy controls after three vaccine doses. Patients with arthritis had lower frequencies of responding T cells after vaccination compared to patients with IBD and controls, but comparable polyfunctional responses. Furthermore, we have shown that CD4 and CD8 T cell responses exhibit different patterns of activation after vaccination and highlight the evolving quality of CD8 T cell responses, characterised by increasing polyfunctionality after breakthrough infections. Importantly, breakthrough infection after vaccination elicits strong T cell responses to both spike and non-spike targets in patients with IBD or arthritis on TNFi therapy. This suggests that hybrid immunity further improves the quality of reactive T cell responses and may increase pathogen clearance and contribute to better long-term clinical protection against severe COVID-19. This study contributes to improved understanding of immunological responses to vaccination and viral infections in patients on TNFi therapies by assessing the distinct dynamics of CD4 and CD8 T cell activation and the quality of their immune responses. A better understanding of T cell responses to hybrid immunity in SARS-CoV-2 is important to inform future vaccination programmes for vulnerable patient groups.

**Implications of all the available evidence**

As cellular immunity is of great importance for modulating the severity of COVID-19, T cell responses are an important metric for protection following vaccination. Polyfunctionality is similar across all patient groups and controls and has been linked with improved clinical outcomes as well as longevity and strength of vaccine responses. Accounting for other underlying conditions and age, our data support the possibility that patients with IBD on TNFi who have received three SARS-CoV-2 vaccine doses may be able follow the general population guidelines for SARS-CoV-2 vaccination in the future.

## Introduction

Patients with inflammatory bowel disease (IBD), arthritis, and other immune-mediated inflammatory diseases treated with tumour necrosis factor inhibitors (TNFi) have impaired humoral immune responses to vaccination,^1^ including against SARS-CoV-2.^2^, ^3^, ^4^, ^5^, ^6^, ^7^ Additionally, levels of antibodies to the receptor-binding domain (RBD) of the spike protein decline more rapidly than in healthy controls.^8^, ^9^, ^10^, ^11^ TNFi therapies are associated with susceptibility to serious infections,^12^^,^^13^ and may lead to an increased risk of COVID-19 breakthrough infection. Many countries now recommend up to six SARS-CoV-2 vaccine doses for individuals on immunosuppressive therapies^14^ and new available vaccines have been modified to reflect the changing circulation of SARS-CoV-2 variants, most recently XBB.1.5.^15^ However, the benefit of additional booster doses in patients on TNFi is unknown.

Neutralising antibodies against SARS-CoV-2, which can block viral entry into cells and prevent infection, are considered a correlate of protection against both infection and disease, although a threshold of protection has not yet been established.^16^ As new SARS-CoV-2 variants escape neutralising antibodies generated after COVID-19 vaccination,^17^ cellular immunity is crucial for long-term protection and viral clearance^16^^,^^18^; mouse models have also shown that T cells can protect against SARS-CoV-2 challenge in an antibody-independent manner.^19^ It has previously been shown that humoral immunity is reduced in patients on TNF inhibitors,^2^^,^^9^^,^^20^ and furthermore appears to wane more quickly over time,^21^ but less is known about cellular immunity post-SARS-CoV-2 vaccination. Prior studies, mainly indirectly assessing T cell responses via cytokine release from peripheral blood mononuclear cells (PBMCs) following up to three vaccine doses, have provided conflicting results regarding differences in responses between patients on TNFi and healthy controls.^20^^,^^22^, ^23^, ^24^ Furthermore, there is a lack of knowledge about the quality, i.e. polyfunctionality, of T cell responses, which can be measured by co-expression of multiple functional markers including cytokines and chemokines.^25^ T cell polyfunctionality has been shown to be associated with improved cellular responses after vaccination in multiple disease models including SARS-CoV-2, HIV, tuberculosis, and numerous other viral, bacterial, and parasitic infections.^25^, ^26^, ^27^, ^28^

A better understanding of polyfunctional T cell responses to hybrid immunity in SARS-CoV-2 is therefore needed to inform future vaccination programmes and to improve our understanding of immunological responses to vaccination and viral infections in patients on TNFi therapies. The objective of this study was to evaluate the magnitude and quality of longitudinal antibody and T cell responses after up to four SARS-CoV-2 vaccine doses and subsequent breakthrough infection in patients with IBD and arthritis on TNFi treatment in order to assess the need for further vaccine boosters in this patient population.

## Methods

### Study and participants

The Norwegian study of vaccine response to COVID-19 (Nor-vaC) is an ongoing prospective, longitudinal observational study (Clinicaltrials.gov, NCT04798625) conducted at two large Norwegian centres for patients with immune-mediated inflammatory diseases: the Department of Gastroenterology at Akershus University Hospital, Lørenskog and the Division of Rheumatology at Diakonhjemmet Hospital, Oslo. The study includes adult patients (aged ≥18 years) with IBD or inflammatory arthritis, including rheumatoid arthritis, spondyloarthritis, and psoriatic arthritis, on immunosuppressive therapies as described previously.^2^ Patients on TNFi therapies diagnosed with Crohn's disease, ulcerative colitis, rheumatoid arthritis, spondyloarthritis, or psoriatic arthritis were identified by hospital records. Health care workers were recruited as healthy controls. Recruitment began prior to the initiation of the national SARS-CoV-2 vaccination program in February 2021. Participants were offered vaccination against SARS-CoV-2 according to recommendations from the national vaccination program by the Norwegian Institute of Public Health, comprised of three primary vaccine doses followed by a fourth booster dose >12 weeks following the third dose (Table 1). Healthy controls received three primary vaccine doses only at this time. Healthy controls with three vaccine doses and a subsequent breakthrough SARS-CoV-2 infection were sampled within 90 days of infection.

**Table 1:** Participant demographics for this study.

Demographics	All patients^a^ (n = 143)	IBD group (n = 118)	Arthritis group (n = 25)	Healthy controls (n = 73)
Age, years	46 (15)	45 (14)	54 (15)	46 (13)
Female	73 (51%)	56 (47%)	17 (68%)	57 (78%)
Male	70 (49%)	62 (53%)	8 (32%)	16 (22%)
Body-mass index, kg/m^2^	27 (6)	28 (6)	27 (6)	–
Current smoker	18 (13%)	14 (12%)	5 (20%)	–
**Diseases**				
Crohn's disease	73 (51%)	73 (62%)	–	–
Ulcerative colitis	45 (32%)	45 (38%)	–	–
Rheumatoid arthritis	9 (6%)	–	9 (36%)	–
Spondyloarthritis	13 (9%)	–	13 (52%)	–
Psoriatic arthritis	3 (2%)	–	3 (12%)	–
**Medication**				
TNF inhibitor, monotherapy	82 (57%)	72 (61%)	10 (40%)	–
TNF inhibitor, combination therapy^b^	61 (43%)	46 (39%)	15 (60%)	–
**TNF inhibitor**				
Infliximab	98 (69%)	73 (62%)	25 (100%)	–
Adalimumab	41 (28%)	41 (34%)	0 (0%)	–
Golimumab	4 (3%)	4 (4%)	0 (0%)	–
**Breakthrough infection**	105 (73%)	94 (80%)	11 (44%)	–
**First vaccine**				
BNT162b2	113 (79%)	99 (84%)	14 (56%)	41 (60%)
mRNA-1273	27 (19%)	18 (15%)	9 (36%)	21 (31%)
ChAdOx1	3 (2%)	1 (1%)	2 (8%)	6 (9%)
**Second vaccine**				
BNT162b2	116 (82%)	100 (85%)	16 (64%)	44 (65%)
mRNA-1273	26 (18%)	17 (15%)	9 (36%)	24 (35%)
**Third vaccine**				
BNT162b2	95 (70%)	73 (65%)	18 (95%)	43 (83%)
mRNA-1273	41 (30%)	40 (35%)	1 (5%)	9 (17%)
Homologous vaccine series	94 (69%)	77 (68%)	15 (65%)	23 (44%)
Heterologous vaccine series^b^	42 (31%)	36 (32%)	8 (35%)	29 (56%)
**Fourth vaccine**				
BNT162b2	43 (45%)	33 (42%)	10 (63%)	–
mRNA-1273	45 (47%)	41 (52%)	4 (25%)	–
Bivalent vaccine^d^	7 (8%)	5 (6%)	2 (12%)	–
Homologous vaccine series	39 (41%)	31 (39%)	9 (56%)	–
Heterologous vaccine series^c^	56 (59%)	48 (61%)	7 (44%)	–

Data are mean (SD) or n (%). Columns show the numbers and demographics of all patients on TNFi treatments (n = 143), only patients with IBD (n = 118), only patients with arthritis (n = 25), and healthy controls (n = 73).

a Patients with T cells sampled at minimum one timepoint.

b TNF inhibitor in combination with azathioprine (n = 37), methotrexate (n = 21), sulfasalazine (n = 2), or prednisolone (n = 1).

c Combination of the following vaccines: CHAdOx1, BNT162b2, mRNA-1273, bivalent vaccine.

d BNT162b2/Omicron BA.1, BNT162b2/Omicron BA.4-5, or mRNA-1273/Omicron BA.1.

**Fig. 1:**
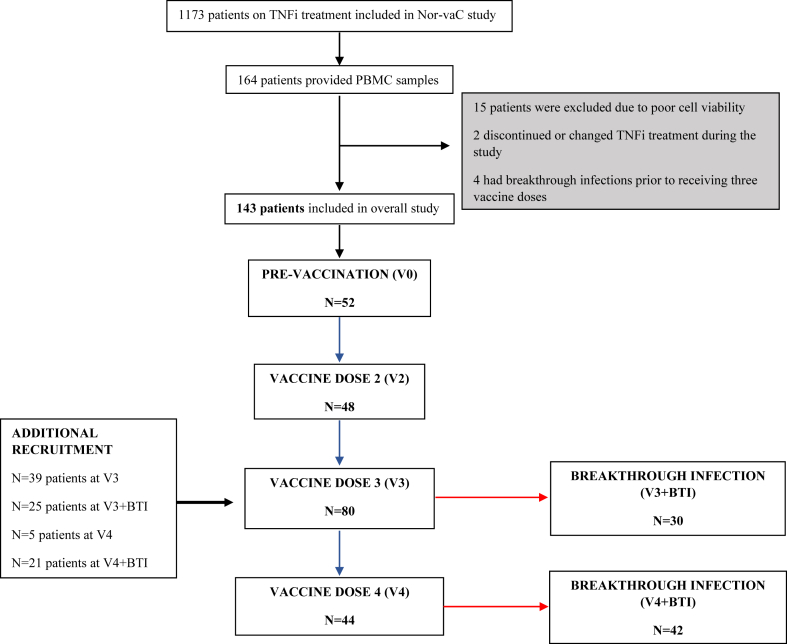
**Study design and patient recruitment**. Longitudinal T cell sampling of patients after sequential SARS-CoV-2 vaccine doses. Throughout the study, additional recipients of third (V3) and fourth (V4) vaccine doses and patients infected with SARS-CoV-2 (breakthrough infections, BTI) were recruited.

Before vaccination, a subset of patients on TNFi from the larger Nor-vaC study and controls were consecutively recruited to a subpopulation that provided serum and PBMC samples at baseline and 2–4 weeks after their second, third and/or fourth vaccine doses (Fig. 1). For feasibility reasons, PBMCs were only collected in a subset of patients followed longitudinally. All relevant samples donated by patients were included. Patients with breakthrough SARS-CoV-2 infections after three or four vaccine doses gave additional PBMC samples 2–4 weeks after infection. Patients who changed or discontinued TNFi treatment during the course of the study, whose PBMC samples had poor viability after cryopreservation and thawing (viability <30%), or who were infected with COVID-19 prior to their third vaccine dose were excluded from analyses (Fig. 1).

### Ethics

The study was approved by the Norwegian Regional Committees for Medical and Health Research Ethics (reference numbers 235424, 135924, REK 2021.233704, and REK 2020.135924). Written informed consent was obtained from all participants.

### Data collection

Demographic data were self-reported and collected at baseline. Data on immunosuppressive medication were collected at the time of vaccination and infection. Sex data were based on national identity number indicating sex. SARS-CoV-2 infections were self-reported by patients, who were reminded every month to report any breakthrough infection, verified by either a PCR test or a rapid antigen test. Data on administered vaccine types were provided from the national database of vaccinations, the Norwegian Immunisation Registry. COVID-19-related hospital admissions were recorded from the Norwegian Patient Registry. Comorbidities were registered from hospital records.

### Frequencies of spike-specific T cell responses: activation-induced marker (AIM) assays

PBMCs were isolated from whole blood collected with sodium citrate and stored in foetal calf serum (FCS) with 10% dimethyl sulfoxide (DMSO) at −150 °C. Cryopreserved PBMCs were thawed in RPMI with 10% FCS and 20 μg/ml DNase I. Dead cells were removed with Dead Cell Removal Kit (Miltenyi Biotec) according to the manufacturer's instructions. Remaining cells were counted and plated at 1–2 x 10^6^ cells/well in 96-well U-bottom plates in culture medium (TexMACS media supplemented with 20 IU/ml IL-2, 1 mM sodium pyruvate (Gibco, Thermo Fisher), 1× MEM NEAA (Gibco), 50 nM 1-thioglycerol and 12 μg/ml gensumycin), rested for 4 h and then stimulated at 37 °C in 5% CO_2_ with SARS-CoV-2 spike peptides (PepTivator SARS-CoV-2 Prot_S and Prot_S Complete, cat. #130-126-701 and #130-127-953). Brefeldin A (Transport Inhibitor, Miltenyi Biotec) was added after 1 h. After 16 h, cells were stained for viability with Live/Dead Fixable Near-IR Dead Cell Stain Kit (cat. #L34976, Thermo Fisher Scientific) at 1:1000 dilution for 20 min at 4 °C, fixed with CytoFix/CytoPerm (BD Biosciences) for 20 min at 4 °C, and stained with fluorescent antibodies (CD3-BV785, AB_2687178, CD8-PerCP-Cy5.5, AB_893422, IL-2-Alexa488, AB_493368, CD137-PE, AB_314783, IFN-γ-Alexa647, AB_493031, CD154-BV510, AB_2563833 (all BioLegend), CD4-PE-Cy5, AB_2833103, Granzyme B-PE-CF594, AB_2737618 (both BD), and TNF-α, AB_469686 (Thermo Fisher)) for 1 h at 4 °C. Cells were acquired on a ZE5 flow cytometer (Bio-Rad). PBMCs from patients with breakthrough infections were also stimulated with SARS-CoV-2 nucleocapsid and membrane peptides (PepTivator SARS-CoV-2 Prot_N, cat. #130-126-699, and Prot_M, cat. #130-126-702, all Miltenyi Biotec). Data were analysed with FlowJo™ v.10.7 Software (BD Life Sciences, RRID:SCR_008520). PBMC samples with <30% viability after cryopreservation and overnight incubation were excluded from the analysis (n = 9). The gating strategy for assessing frequencies of activated CD40L + TNF-α+ CD4 and IFN-γ+ TNF-α+ CD8 T cells is shown in Appendix p7, Supplementary Figure S1. Positive T cell responses were defined as responses >0.001% after subtracting the unstimulated control. Responses below this level were assigned as 0.0005%.

### Quality of T cell responses (polyfunctionality)

Spike-specific polyfunctionality (expression of two or more of CD40L/CD154, CD137/4-1BB, IFN-γ, TNF-α, and IL-2) was analysed with a Bayesian statistical framework using the combinatorial polyfunctionality analysis of single cells (COMPASS) algorithm.^27^ The polyfunctionality score (PFS) was calculated from COMPASS posterior probabilities for spike-specific T cell responses (Supplementary Figure S2, Appendix p8 and 9). Preprocessing was done using the R packages PeacoQC (version 1.11.2) and gating with the flowCore (v. 2.15.3) and flowDensity deGate (v. 1.37.3) functions. Preprocessed CD4 and CD8 positive events were separated and down sampled to 20000 events per file and subjected to quantitative analysis and COMPASS, which summarizes the participant-level responses with the functionality score (FS) and the PFS quantifying the quality of a participant's immune response. FS is the estimated proportion of spike-specific subsets identified across all possible functional subsets and PFS is the weighted FS by their degree of functionality. In this study, expression of CD40L, 4-1BB/CD137, IFN-γ, TNF-α, and IL-2 were included in the CD4 and CD8 T cells analysis with all default settings.

### Antibody analyses

IgG to the receptor-binding domain (RBD) of SARS-CoV-2 spike protein in serum was measured in WHO adjusted units (BAU/ml) using an in-house bead-based method calibrated against the Roche Elecsys Anti-SARS-CoV-2 S assay and a micro-neutralisation assay at the Department of Immunology at Oslo University Hospital.^29^ Anti-RBD levels were calculated for Wuhan-Hu-1 and BA.1 (omicron) strains. Levels >5 BAU/ml were considered positive. The dynamic range was 5–100,000 BAU/ml. Anti-nucleocapsid IgG levels were measured at the same time points and used to track infection history in patients; the threshold for a positive response was >7 arbitrary units/ml (AU/ml).

### Statistics

Data were analysed using GraphPad Prism version 9.0.0, Stata v.17 (StataCorp), and R (R version 3.4.0). T cell analyses by dependent samples sign tests were adjusted for multiple comparisons by controlling the false discovery rate (FDR). All statistical analyses reported in the text and figures are adjusted p-values. Dependent samples sign tests were used wherever the number of paired samples was equal or greater than n = 30. Age, vaccine type, diagnosis, and treatment (mono- or combination therapy) on antibody and cellular responses were adjusted for by multivariable linear regression wherever longitudinally paired samples were not available using log (antibody) and log (T cell responses), with the exception of CD4 PFS responses, which were not log transformed. Goodness of fit was visually examined using diagnostic plots in R. Results are reported as the multiplicative factor for every one-unit increase in the independent variable, except for CD4 PFS responses, which are reported as additive factors. Values reported in the text are given as median (IQR); regression analysis beta coefficients are presented as estimate (95% CI). Relationships between antibody and cellular responses were fitted using local polynomial regression fitting (loess), which were generated in R using the default parameters in the geom_smooth () function. As the Nor-vaC study was an observational study aiming to assess several aspects of vaccine responses, no prior sample size evaluation was performed and all eligible patients at the two clinical centres were invited to participate. A directed acyclic graph (DAG) (Supplementary Figure S3, Appendix p10) shows the factors considered in this study using the web application DAGitty v.3.1.^30^ The minimal sufficient adjustment set contains age, arthritis, hypertension, IBD, smoking, and vaccine type for estimating the total effect of TNFi treatment on immune responses after vaccination. All available PBMC samples were included in the analysis unless excluded for reasons stated above.

### Role of funders

The funders of the study had no role in the design or conduct of the study, data collection, analysis, interpretation, writing of the manuscript, or decision to submit the manuscript for publication.

## Results

Between March 2, 2021, and December 20, 2022, 1173 patients with IBD or arthritis on TNFi therapy were included in the Nor-vaC study and provided post-immunisation serology at least at one timepoint. Of these, 143 patients (82 (57%) on TNFi monotherapy and 61 (43%) on combination therapy) provided post-vaccination PBMC samples for spike-specific CD4 and CD8 T cell responses 2–4 weeks after up to four SARS-CoV-2 vaccine doses or a breakthrough infection (Fig. 1). The median age was 48 years (IQR 33–57). 73 (51%) were women and 70 (49%) were men. A total of 118 patients had IBD (73 (62%) had Crohn's Disease and 45 (38%) had ulcerative colitis) and 25 patients had arthritis (9 (36%) had rheumatoid arthritis, 13 (52%) spondyloarthritis, and 3 (12%) psoriatic arthritis). The control group included 73 healthy individuals who received up to three vaccine doses. Baseline characteristics for patients and healthy controls, including vaccine distribution, are presented in Table 1.

Breakthrough infection was reported by 105 patients, of whom 72 provided T cells pre- and post-infection (30 patients after the third vaccine dose and 42 patients after the fourth dose (Fig. 1)). In total, 94% of the reported infections occurred in the period between January 1, 2022, and December 20, 2022, while omicron strains BA.1, BA.2, and BA.5 were circulating in Norway. None of the patients with breakthrough infections required hospitalisation and no COVID-19-related deaths occurred during the observation period. Patients did not receive prophylactic or post-exposure antiviral medications.

**Fig. 2:**
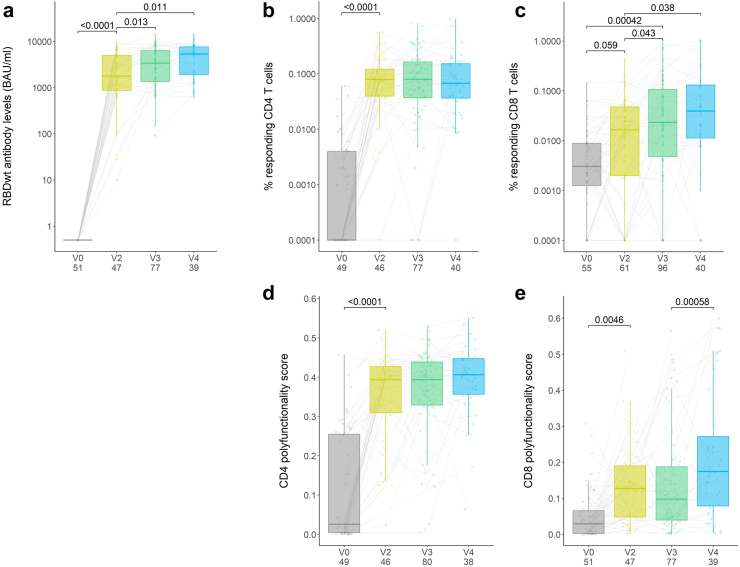
**Humoral and cellular responses in patients on TNFi treatments after vaccination**. (a) Anti-Wuhan-Hu-1 receptor-binding domain (RBDwt) IgG concentrations at baseline (V0) and after two (V2), three (V3), and four (V4) vaccine doses. Responses of under 5 binding antibody units (BAU)/ml were considered negative. (b and c) Frequency of spike-specific CD4 and CD8 T cell responses for all patients on TNFi treatments at baseline and after two, three, and four vaccine doses. (d and e) Polyfunctionality score for spike-specific CD4 and CD8 T cell responses. Box plots indicate median and interquartile range (IQR), whiskers indicate 1.5 × IQR extending from Q1 and Q3. Numbers per group are indicated on plots. Grey lines between points indicate paired samples. Points without lines indicate non-longitudinal samples which were not used for the statistics. Statistical analyses were calculated by dependent samples sign tests with false discovery rate (FDR) correction on the same individuals at different time points. All p-values are adjusted for multiple comparisons. PFS, polyfunctionality score.

All patients seroconverted after two vaccine doses (p < 0.0001, sign test). Additional vaccine doses further enhanced antibody responses (antibody levels after two doses vs. four doses were 1771 (795–5207) vs. 5327 (1752–7813) BAU/ml, p = 0.011) (Fig. 2a). Spike-specific CD4 T cell responses were detected in 44/46 (95.6%) of patients after two vaccine doses (baseline vs. second dose 0.0001% (0.0001–0.004) vs. 0.080% (0.039–0.140), p < 0.0001, sign test). An additional third and fourth dose in these patients did not further increase the CD4 responses compared to the second vaccine dose (third dose 0.079% (0.034–0.170), p = 1.00; fourth dose 0.068% (0.034–0.160), p = 0.43) (Fig. 2b). By contrast, the magnitude of CD8 T cell responses continued to increase with additional vaccine doses up to four doses (baseline, second, third, and fourth doses were 0.003% (0.001–0.009), 0.014% (0.002–0.029), 0.021% (0.004–0.079), and 0.039% (0.010–0.140)) and showed an increase from baseline to second dose (p = 0.059) and from second to third dose (p = 0.043, all sign tests) (Fig. 2c).

Consistent with the quantity of responding cells in these patients, the quality of spike-specific polyfunctional CD4 T cell responses plateaued after two vaccine doses (PFS at baseline vs. second dose, 0.03 (0.004–0.26) vs. 0.39 (0.31–0.43), p < 0.0001) (Fig. 2d and Supplementary Figure S2a, Appendix p8). Polyfunctional CD8 T cell responses continued to increase with additional vaccination (PFS at baseline vs. second dose, 0.03 (0.003–0.07) vs. 0.13 (0.05–0.19), p = 0.0046, third vs. fourth dose, 0.10 (0.04–0.19) vs. 0.17 (0.08–0.27), p = 0.00058, all sign tests) (Fig. 2e and Supplementary Figure S2b, Appendix p9).

**Fig. 3:**
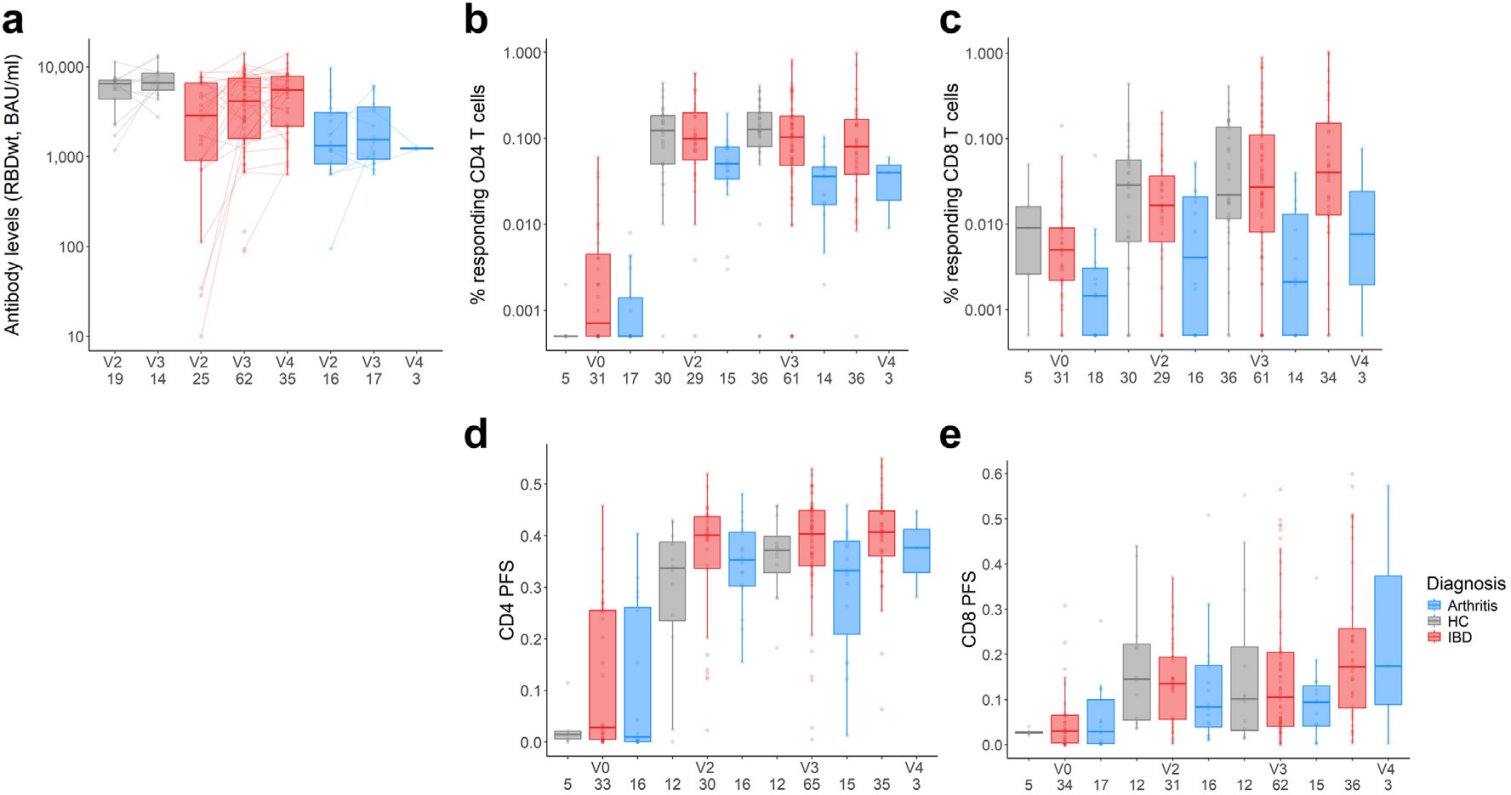
**Immune responses after vaccination vary with diagnosis**. (a) Anti-RBDwt IgG titres after two, three, and four vaccine doses in healthy controls (grey), patients with IBD (red), and patients with arthritis (blue). Lines indicate paired samples over time. (b and c) CD4 T cell spike-specific responses showing percentages of responding cells and polyfunctionality score at baseline and after two, three, and four vaccine doses in healthy controls, patients with IBD, and patients with arthritis. (d and e) CD8 T cell spike-specific responses showing percentages of responding cells and polyfunctionality score at the same four time points for the different diagnosis groups. Box-and-whisker plots indicate median, IQR and 1.5 × IQR from Q1 and Q3. Numbers per group are indicated on plots. V0, baseline; V2, vaccine dose two; V3, vaccine dose three; V4, vaccine dose four; HC, healthy controls; IBD, inflammatory bowel disease; PFS, polyfunctionality score. Statistical comparisons by multivariable linear regression analysis are described in the results.

Patients with IBD or arthritis had lower humoral immune responses than healthy controls following both the second and third vaccine doses (anti-RBDwt IgG for IBD, arthritis and controls after two doses, 2873 (809–6659), 1322 (754–3347), and 6472 (4358–7103) BAU/ml; after three doses, 3954 (1484–7477), 1454 (891–3680), and 6280 (4907–8511) BAU/ml) (Fig. 3a). However, when controlling for age, treatment, and vaccine type using multivariable linear regression analysis, we found that age and vaccine type (receiving three doses of mRNA-1273 or a combination of mRNA vaccines, compared to three doses of BNT162b2) accounted for the majority of these differences (estimated regression coefficient of age, 0.98 (95% CI 0.97–0.99), p = 0.012; three doses of mRNA-1273, 2.59 (1.21–5.53), p = 0.016; heterologous vaccination, 1.73 (1.06–2.80), p = 0.030) (Supplementary Table S1, Appendix p2). After three vaccine doses we found no impact of an IBD diagnosis on T cell responses compared to healthy controls by multivariable linear regression, and CD4 and CD8 T cell responses reached similar levels in both patients with IBD and healthy controls (CD4 T cells, 0.11% (0.052–0.20) vs. 0.13% (0.080–0.20); CD8 T cells, 0.031% (0.008–0.11) vs. 0.022% (0.012–0.14), Fig. 3b and c). Likewise, patients with IBD demonstrated comparable T cell polyfunctionality to healthy controls in both CD4 and CD8 T cells (CD4 PFS 0.40 vs. 0.37 and CD8 PFS 0.11 vs. 0.10, Fig. 3d and e) (estimated regression coefficients are summarised in Supplementary Table S1, Appendix p2).

Multivariable linear regression indicated that patients with arthritis had lower CD8 T cell responses than the healthy control group (frequency of responding CD8 after three vaccine doses 0.002% (0.0001–0.016), regression coefficient 0.11 (95% CI 0.02–0.53), p = 0.0072) (Fig. 3c). We also observed a positive impact of age on CD8 responses (1.04 (95% CI 1.01–1.07), p = 0.021). However, we did not find any significant impact of arthritis on the percentage of responding CD4 T cells (median response after three doses 0.029% (0.011–0.047)). Furthermore, both spike-specific polyfunctional CD4 and CD8 T cell responses after three vaccine doses in patients with arthritis were comparable to healthy controls and patients with IBD (PFS of patients with IBD, arthritis, and healthy controls for CD4 responses, 0.40 (0.34–0.45) vs. 0.33 (0.15–0.40) vs. 0.37 (0.30–0.43); for CD8 responses, 0.10 (0.04–0.21) vs. 0.09 (0.04–0.14) vs. 0.10 (0.02–0.30)) (Fig. 3d and e; Supplementary Table S1, Appendix p2).

In the total group of patients on TNFi, we found a positive association between CD4 and CD8 T cell responses (Supplementary Figure S4a), and between antibody levels and both CD4 and CD8 responses after three vaccine doses (Supplementary Figure S4b, Appendix p11). After adjusting for diagnosis, age, and mono- or combination therapy, individuals in all three groups vaccinated with a heterologous prime-boost vaccination schedule (either two doses of BNT162b2 and a third dose of mRNA-1273 or vice versa) had significantly higher antibody levels, CD4, and CD8 T cell responses after three vaccine doses than those who received homologous vaccination (three doses of either mRNA vaccine) (Supplementary Table S1, Appendix p2). We found no significant differences in T cell responses between patients using adalimumab or infliximab, or between patients receiving monotherapy compared to combination therapy after primary vaccination (Supplementary Table S1 and Supplementary Figure S5, Appendix p2 and 12).

**Fig. 4:**
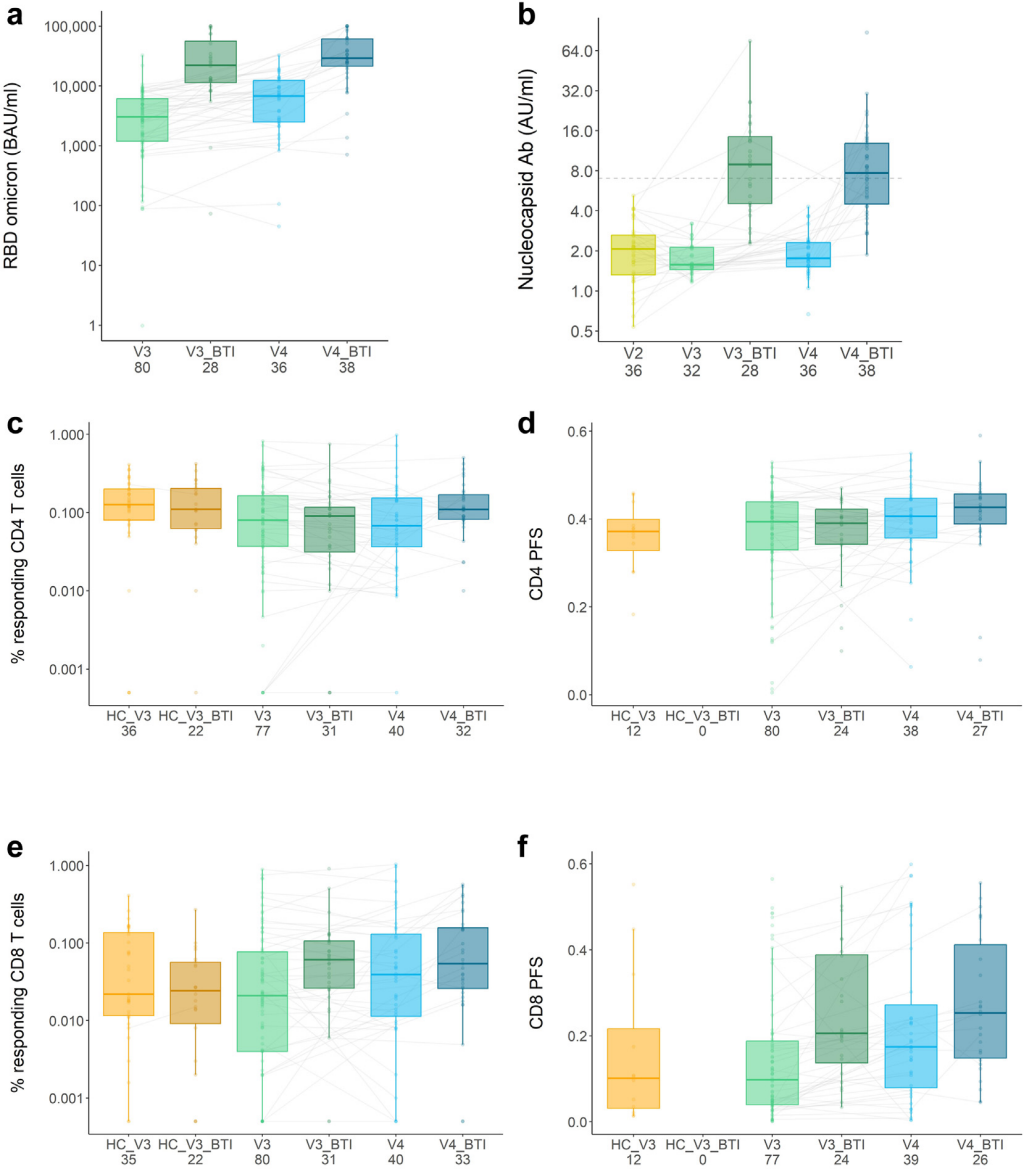
**Immune responses following SARS-CoV-2 vaccination and breakthrough infection after vaccine doses three and four in patients on TNFi treatment**. (a) IgG responses to RBD omicron before and after breakthrough infection for patients with three or four vaccine doses. (b) IgG responses to nucleocapsid after two, three, and four vaccine doses and after breakthrough infection. (c and d) CD4 T cell spike-specific responses showing percentages of responding cells and polyfunctionality score before and after SARS-CoV-2 infection after three or four vaccine doses. (e and f) CD8 T cell spike-specific responses showing percentages of responding cells and polyfunctionality score before and after SARS-CoV-2 infection after three or four vaccine doses. Box-and-whisker plots indicate median, IQR and 1.5 × IQR from Q1 and Q3. Numbers per group are indicated on plots. Blue and green shades indicate patients on TNFi, orange plots indicate healthy controls. Grey lines between points indicate paired samples. Points without lines indicate non-longitudinal samples. Polyfunctionality data for healthy controls after breakthrough infection are not available (d and f). HC, healthy control; V2, vaccine dose two; V3, vaccine dose three; V4, vaccine dose four; BTI, breakthrough infection; RBD, receptor-binding domain; PFS, polyfunctionality score. Statistical comparisons by multivariable linear regression analysis are described in the results.

Breakthrough infection significantly increased IgG levels of anti-RBD antibodies (anti-RBD omicron BA.1 before and after infection for three doses was 3051 (1153–6155) vs. 22243 (10546–67862) BAU/ml, and 6803 (2402–12924) vs. 29134 (20382–62388) BAU/ml for four doses) (Fig. 4a and Supplementary Table S2, Appendix p3). Analysis by multivariable linear regression showed that this increase was primarily driven by breakthrough infection (estimated regression coefficients were 5.45 (95% CI 2.86–10.38), p < 0.0001, and 4.73 (2.72–8.26), p < 0.0001, respectively). Likewise, infection drove anti-nucleocapsid antibody responses (before infection, at three and four vaccine doses nucleocapsid antibody levels were 1.48 (1.44–2.15) and 1.76 (1.49–2.32) AU/ml respectively and after infection 8.91 (4.44–15.25) and 7.68 (4.33–13.14) AU/ml) (Fig. 4b). However, the frequencies of spike-specific T cell responses were comparable before and after breakthrough infection in patients who had received three and four vaccine doses. In patients with three vaccine doses, CD4 T cell frequencies were 0.079% (0.034–0.167) before and 0.090% (0.028–0.122) after infection; CD8 T cell frequencies were 0.021% (0.004–0.079) vs. 0.061 (0.025–0.108). In patients who had received four vaccine doses, CD4 T cell frequencies were 0.068% (0.034–0.162) before and 0.110% (0.080–0.177) after infection, and CD8 T cell frequencies were 0.039% (0.010–0.145) vs. 0.054% (0.025–0.166) (Fig. 4c and e). Multivariable linear regression did not show an impact of diagnosis, age, or treatment type on CD4 responses before and after infection, although heterologous primary vaccination had some impact on increased responses after three vaccine doses (regression coefficient 2.33 (95% CI 1.17–4.67), p = 0.018) but not after four. T cell responses after a breakthrough infection were similar in healthy controls and patients on TNFi after three vaccine doses (healthy control CD4, 0.110% (0.063–0.204); CD8, 0.024% (0.009–0.057)).

Interestingly, CD8 polyfunctional responses increased after breakthrough infection. The median polyfunctionality score after three vaccine doses was 0.10 (0.04–0.19) before infection vs. 0.21 (0.12–0.39) after infection (Fig. 4f). Multivariable linear regression indicated that this was due to a breakthrough infection (estimated regression coefficient 2.26 (95% CI 1.20–4.25), p = 0.013) rather than diagnosis, age, treatment, or vaccine type (Supplementary Table S2, Appendix p3). After four vaccine doses, the polyfunctionality score was 0.17 (0.07–0.30) vs. 0.25 (0.14–0.44)) before and after infection and this was not significantly affected by any of the tested variables. CD4 polyfunctional responses did not significantly increase after infection (Fig. 4d). Following a breakthrough infection, the majority of patients had nucleocapsid-specific (32/44 (73%) patients) and membrane-specific CD4 responses (29/44 (66%) patients). Likewise, we detected nucleocapsid-specific (36/44 (82%) patients) and membrane-specific CD8 responses (25/44 (57%) patients). Infections induced non-spike T cell responses in patients with IBD or arthritis (Supplementary Figure S6, Appendix p13). Nucleocapsid and membrane responses showed limited association with spike-specific responses (Supplementary Figure S7, Appendix p13).

## Discussion

In this observational study encompassing patients with IBD or arthritis on TNFi treatment, patients with IBD demonstrated strong T cell responses within four weeks after two to four doses of SARS-CoV-2 mRNA vaccines. Although patients on TNFi treatments had low humoral immune responses, patients with IBD and healthy controls had comparable T cell responses after both two and three vaccine doses. The frequency and quality of spike-specific CD4 T cell responses plateaued after two vaccine doses whereas CD8 T cell responses continued to improve in quantity and quality up to four doses or immunising events. Immune responses in patients on TNFi were further enhanced after breakthrough infections as indicated by the induction of T cells reactive to peptides from non-spike SARS-CoV-2 antigens and an increase in polyfunctional spike-specific CD8 T cell responses.

Previous research has yielded conflicting results regarding the attenuation of T cell responses in patients with IBD undergoing TNFi therapies after receiving two or three doses of SARS-CoV-2 vaccine.^20^^,^^22^^,^^24^ This study shows that patients with IBD had comparable T cell responses to those of healthy individuals following three vaccine doses. We also demonstrated that the responses observed after vaccination and infection were notably polyfunctional, indicating activity against severe COVID-19 disease.^25^, ^26^, ^27^^,^^31^ These robust T cell responses support the finding that patients on TNFi therapies were not significantly more likely to be hospitalised or die from COVID-19 than the general population prior to vaccination.^32^ Another recent study on our patient cohort found that the incidence and outcome of COVID-19 was the same in patients on TNFi as in healthy controls after vaccination.^33^ Although studies show conflicting evidence, data suggest that vaccinated patients with IMID were not more likely to have a SARS-CoV-2 omicron infection than healthy controls.^34^ We found no differences in cellular responses amongst patients with IBD using TNFi monotherapy or combination therapy with azathioprine or methotrexate. We observed slightly higher frequencies of responding T cells at the third vaccine timepoint in individuals who received a heterologous vaccine combination compared to homologous vaccination, which is in line with observations from other studies,^35^ but responses were similar overall including the quality of the vaccine response as measured by polyfunctionality.

In our study, patients with arthritis had slightly lower frequencies of responding CD8 T cells than both patients with IBD and healthy controls after vaccination and this difference remained significant after adjustments for age, treatment, and vaccine type, although the responding cells showed similar levels of polyfunctionality. Notably, this difference was ameliorated after a subsequent breakthrough infection, and patients with arthritis made new T cell responses to non-spike peptides comparable to patients with IBD. The number of individuals with arthritis was small, and these findings should be interpreted with caution.

In agreement with two recent studies in healthy individuals,^23^^,^^26^ we found that the quality of T cell responses, as measured by expression of cytokines (IFN-γ, TNF-α, and IL-2) and activation markers (CD40L and CD137) and described here as polyfunctionality, continued to improve after further immunising events. IFN-γ and TNF-α mediate clearance and killing of pathogens, while IL-2 drives proliferation and expansion of T cell populations, and their co-expression by antigen-specific cells is widely considered to be an important marker of a protective immune response after vaccination.^25^^,^^27^^,^^28^ Distinguishing between the CD4 and CD8 immune compartments and the dynamics of activation after vaccination and infection can provide greater nuance for understanding how protective cellular responses are mediated. Our data suggest that the CD4 T cell response is rapidly induced after two vaccine doses and correlates with antibody responses, whereas CD8 T cell responses take more immunising events to develop.

Although infection induced cellular responses against non-spike targets, the magnitude of the spike-specific T cell response peaked in most individuals after four immunising events, whether vaccine or infection derived, suggesting that cellular responses cannot be increased indefinitely. Whether polyfunctionality further increases with additional vaccines or infections cannot be determined from the present study. Our approach to measuring polyfunctionality using the COMPASS method^27^ has allowed us to describe the multiple subsets of responding polyfunctional cells more comprehensively instead of measuring individual cytokine production or manually selecting cells of interest. This analysis also highlights how T cell responses continue to evolve with further vaccine doses despite the stable frequency of responding cells and how the expression of distinct combinations of cytokines and activating markers changes with time. Although we cannot suggest a threshold of protection for T cell responses, polyfunctionality has been associated with clinical outcome after both infection and vaccination^25^^,^^27^ and therefore the robust polyfunctional T cell responses seen in both groups of patients in this study, as well as the lack of adverse outcomes after breakthrough infection, suggest that these individuals have cellular responses that may mediate protection against severe disease as described by others.^26^^,^^31^

In healthy individuals, protective immunity against the omicron variant is stronger after hybrid immunity than after vaccination only.^36^ Although we did not assess cellular responses to other SARS-CoV-2 variants, this study demonstrated that hybrid immunity after breakthrough infection drives immune responses to nucleocapsid and membrane antigens and provides natural boosting of the spike-specific response. However, antibody titres wane over time and, to date, new viral variants have generally escaped neutralisation by vaccine-derived antibodies.^37^ New vaccine formulations may therefore be valuable for boosting the humoral response and reducing the incidence of infection as both neutralising antibodies and antibodies with other effector functions have important roles in viral control and clearance.^16^

One major strength of this study is the longitudinal study design of this cohort with follow up over 22 months, with blood sampling and questionnaires that enabled successive registration of breakthrough infections and changes in medication. This allowed for detection of immune responses in individuals over time and permitted us to distinguish between the roles of vaccination and hybrid immunity. Furthermore, we distinguished between CD4 and CD8 T cell responses and measured cytokine production and activation marker expression at a single-cell level, rather than relying on more indirect cytokine release assays. This allowed us to determine spike-specific activation and the varying degrees of polyfunctionality in these cell populations at different time points and chart the response to sequential vaccinations and infection.

Limitations of this study include a lack of assessment of broader T cell responses after vaccination with bivalent vaccines, including the BA.1 or BA.4/5 strains. Additionally, T cells were only stimulated with peptides against the original Wuhan-Hu-1 spike protein, limiting our ability to gauge responses against omicron or other newer variants. Further studies are needed to assess whether bivalent vaccines will add to or improve T cell immunity any further in this patient group. This cohort also had limited numbers of paired samples before and after breakthrough infections and our multivariable linear regressions may not have accounted for all confounding variables. Of note, our study included young people with IBD in their mid-forties with a generally low prevalence of comorbidities,^38^ so the findings made here may not be predictive of immune responses in older patients.^14^

In conclusion, this study adds a comprehensive assessment of T cell responses to both vaccination and breakthrough infection in patients on TNFi treatments in a real-world setting in the omicron era. These results indicate that patients with IBD on TNFi treatments who have received at least three SARS-CoV-2 vaccine doses have robust T cell responses comparable to the general population. Our study adds evidence that, in the absence of other risk factors, this group may in future be able to follow the general recommendations for COVID-19 vaccines. This study is highly relevant to the large number of patients on TNFi therapy and can help to guide treatment and vaccine recommendations as the next stage of dealing with the SARS-CoV-2 pandemic evolves.

## Contributors

GLG, KKJ, SWS, LAM, JTV, FLJ, SM conceived and designed the study. LAM, GLG, KKJ, SM supervised the study. ASW, KHB, HSØ, GS, IFK, IJ, IEC, KPL, ATT, JS contributed to data acquisition and management. AC, FLJ performed the humoral analyses. ASW, GS, IFK, SEJ, JRO, VC, HK performed the cellular analyses. ASW, KHB, SB, UCN, ABK contributed with statistical analysis. ASW, SB, ABK, SM had full access to all the data in the study and take responsibility for the integrity of the data and the accuracy of the data analysis. ASW, KHB, HSØ, SWS, GLG, KKJ, SM drafted the manuscript. KPL, JRO, VC, GG, HK, TKK, EAH, SAP, JJ contributed with administrative and technical support. JTV, FLJ, LAM, GLG, KKJ, SM obtained funding for this study. All authors contributed to interpretation and analysis of the data. All authors revised the manuscript and approved the final submitted version.

## Data sharing statement

A de-identified patient data set can be made available to researchers upon reasonable request. The data will only be made available after submission of a project plan outlining the reason for the request and any proposed analyses and will have to be approved by the Nor-vaC steering group. Project proposals can be submitted to the corresponding authors. Data sharing will have to follow appropriate regulations.

## Declaration of interests

KHB reports funding from Akershus University Hospital and speaker bureaus for Janssen-Cilag. TKK reports grants from AbbVie, BMS, Galapagos, Novartis, Pfizer, UCB, speakers' bureaus from Grünenthal, Janssen, Sandoz, consultant fees from AbbVie, Gilead, Janssen, Novartis, Pfizer, Sandoz, UCB, and participation on advisory board for AbbVie. JJ reports grants from Boehringer-Ingelheim, speakers bureaus from AbbVie/Abbott, Bristol-Myers, Squibb, Galapagos, Gilead, Janssen, Pfizer, Roche, Sandoz, Takeda, consultant fees from AbbVie/Abbott, Pfizer, and participation in advisory board for AbbVie/Abbott, Bristol-Myers Squibb, Galapagos, Gilead, Janssen, Pfizer, Roche, Sandoz, Takeda. LAM reports funding from KG Jebsen foundation, support for infrastructure and biobanking from the university of Oslo and Oslo University Hospital, grants from the Coalition of Epidemic Preparedness Innovations (CEPI), speakers' bureaus from Incyte, Janssen, and expert testimony for Norwegian Medicines Agency. EAH reports speakers' bureaus from Pfizer, UCB, Novartis, and consulting fees from Abbvie, Pfizer, Eli Lilly. GG reports speaker bureaus from AbbVie/Abbott, Galapagos, Pfizer, UCB, participation in advisory board from AstraZeneca, Janssen, Moderna, Seqirus. and consulting fees from The Norwegian System of Patient Injury Compensation. JTV reports grant from CEPI. FLJ reports funding from South-East Health Authorities in Norway, CEPI and Oslo University Hospital. GLG reports speakers' bureaus from AbbVie/Abbott, Galapagos, Pfizer, UCB, and participation in advisory board from AbbVie/Abbott, Galapagos, Pfizer, UCB, Novartis. KKJ reports speakers’ bureaus from Bristol-Myers Squibb and Janssen, and participation in advisory board for AstraZeneca and IPSEN. SWS reports participation in advisory board for AstraZeneca. ASW reports travel grant of GBP 250 from the British Society of Immunology to support travelling to the BSI Congress 2023 (4–7th Dec 2023) and presenting a poster with some of this data. HSØ, SB, GS, IFK, IJ, UCN, ABK, IEC, SEJ, KPL, AC, ATT, JS, SAP, HK, and SM report nothing to disclose.
