# Supplementary material for: T cell responses to repeated SARS-CoV-2 vaccination and breakthrough infections in patients on TNF inhibitor treatment: a prospective cohort study
Source: eBioMedicine. 2024 Sep 10;108:105317. doi: 10.1016/j.ebiom.2024.105317 (PMC11416219; doi:10.1016/j.ebiom.2024.105317)

**Supplementary appendix**

**Supplement to: Wolf AS, Bjørlykke KH, Ørbo HS, et al.** **T cell responses to repeated SARS-CoV-2 vaccination and breakthrough infections in patients on TNF inhibitor treatment: a prospective cohort study**

**Table of contents**

[**Section 1. Supplementary tables** 2](#_Toc171685431)

[Supplementary table 1. Summary of multiple regression results: diagnosis groups 2](#_Toc171685432)

[Supplementary table 2. Summary of multiple regression results: after breakthrough infection 3](#_Toc171685433)

[**Section 2. Supplementary figures** 7](#_Toc171685434)

[Supplementary figure 1. Flow cytometry gating strategy 7](#_Toc171685435)

[Supplementary figure 2. COMPASS heatmap showing mean posterior probabilities of spike-specific responses 8](#_Toc171685436)

[Supplementary figure 3. Directed acyclic graph for study design 10](#_Toc171685437)

[Supplementary figure 4. Associations between T cell responses and humoral responses 11](#_Toc171685438)

[Supplementary figure 5. Effect of TNFi treatment on vaccine responses 12](#_Toc171685439)

[Supplementary figure 6. Non-spike responses after breakthrough infection by diagnosis group 13](#_Toc171685440)

[Supplementary figure 7. Nucleocapsid and membrane responses are not associated with spike-specific responses after breakthrough infections 13](#_Toc171685441)

# **Section 1. Supplementary tables**

## **Supplementary table 1. Summary of multiple regression results: diagnosis groups**

| Table 1: multiple regression for comparison of diagnosis groups | | |
| --- | --- | --- |
| Variables | Estimated regression coefficient (95% CI) | Pr(>\|t\|) |
| Wuhan RBD levels |  |  |
| (Intercept) | 9931 (3235, 30408) | <0·0001 |
| Diagnosis - IBD | 0·70 (0·33, 1·49) | 0·36 |
| Diagnosis - Arthritis | 0·57 (0·24, 1·37) | 0·22 |
| Age (years) | 0·98 (0·97, 0·99) | 0·012 |
| Combination therapy | 0·66 (0·42, 1·02) | 0·064 |
| Vaccine type - mRNA-1273 | 2·59 (1·21, 5·53) | 0·016 |
| Vaccine type - heterologous | 1·73 (1·06, 2·80) | 0·030 |
| Percentage CD4 responses |  |  |
| (Intercept) | 0·055 (0·01, 0·28) | 0·0010 |
| Diagnosis - IBD | 1·26 (0·45, 3·52) | 0·66 |
| Diagnosis - Arthritis | 0·77 (0·17, 3·46) | 0·73 |
| Age (years) | 0·99 (0·96, 1·01) | 0·32 |
| Combination therapy | 1·29 (0·51, 3·27) | 0·59 |
| Vaccine type - mRNA-1273 | 1·86 (0·44, 7·94) | 0·40 |
| Vaccine type - heterologous | 3·29 (1·38, 7·85) | 0·0087 |
| Percentage CD8 responses |  |  |
| (Intercept) | 0·002 (0·0003, 0·01) | <0·0001 |
| Diagnosis - IBD | 1·90 (0·62, 5·82) | 0·27 |
| Diagnosis - Arthritis | 0·11 (0·02, 0·52) | 0·0072 |
| Age (years) | 1·04 (1·01, 1·07) | 0·021 |
| Combination therapy | 0·39 (0·14, 1·06) | 0·068 |
| Vaccine type - mRNA-1273 | 1·79 (0·37, 8·59) | 0·47 |
| Vaccine type - heterologous | 2·88 (1·11, 7·43) | 0·032 |
| CD4 polyfunctionality (PFS)† |  |  |
| (Intercept) | 0·22 (0·1, 0·34) | <0·0001 |
| Diagnosis - IBD | 0·06 (-0·03, 0·15) | 0·17 |
| Diagnosis - Arthritis | -0·02 (-0·12, 0·08) | 0·70 |
| Age (years) | 0·001 (0, 0·003) | 0·14 |
| Combination therapy | -0·01 (-0·06, 0·03) | 0·56 |
| Vaccine type - mRNA-1273 | 0·10 (0·03, 0·18) | 0·010 |
| Vaccine type - heterologous | 0·10 (0·05, 0·15) | 0·00028 |
| CD8 polyfunctionality |  |  |
| (Intercept) | 0·10 (0·02, 0·59) | 0·013 |
| Diagnosis - IBD | 1·46 (0·41, 5·14) | 0·56 |
| Diagnosis - Arthritis | 0·86 (0·20, 3·66) | 0·83 |
| Age (years) | 0·99 (0·97, 1·02) | 0·59 |
| Combination therapy | 0·66 (0·34, 1·30) | 0·23 |
| Vaccine type - mRNA-1273 | 0·68 (0·22, 2·10) | 0·51 |
| Vaccine type - heterologous | 1·02 (0·49, 2·13) | 0·96 |

† Multiple regression analysis was carried out with log transformed data for all values except for CD4 PFS, which was carried out with non-log transformed data. Estimated effects for CD4 PFS are therefore additive.

## **Supplementary table 2. Summary of multiple regression results: after breakthrough infection**

| Table 2: multiple regression for comparison of responses after infection | | |
| --- | --- | --- |
| Variables | Estimated regression coefficient (95% CI) | Pr(>\|t\|) |
| Omicron RBD levels – three vaccine doses |  |  |
| (Intercept) | 9527 (3147, 28840) | <0·0001 |
| Infection | 5·45 (2·86, 10·38) | <0·0001 |
| Diagnosis - Arthritis | 0·92 (0·46, 1·85) | 0·83 |
| Age (years) | 0·97 (0·95, 0·99) | 0·0035 |
| Hypertension | 1·03 (0·50, 2·15) | 0·93 |
| Smoking | 1·56 (0·59, 4·15) | 0·37 |
| Combination therapy | 0·57 (0·33, 1·00) | 0·053 |
| Vaccine type - mRNA-1273 | 3·26 (1·21, 8·79) | 0·022 |
| Vaccine type - heterologous | 1·88 (1·04, 3·40) | 0·038 |
| Omicron RBD levels – four vaccine doses |  |  |
| (Intercept) | 46451 (12647, 170608) | <0·0001 |
| Infection | 4·73 (2·72, 8·26) | <0·0001 |
| Diagnosis - Arthritis | 0·34 (0·12, 0·94) | 0·041 |
| Age (years) | 0·96 (0·94, 0·98) | 0·00024 |
| Hypertension | 0·91 (0·45, 1·82) | 0·78 |
| Smoking | 1·72 (0·64, 4·61) | 0·29 |
| Combination therapy | 0·83 (0·47, 1·47) | 0·52 |
| Vaccine type - mRNA-1273 | 1·72 (0·68, 4·37) | 0·26 |
| Vaccine type - heterologous | 1·14 (0·64, 2·02) | 0·66 |
| Nucleocapsid Ab levels – three vaccine doses |  |  |
| (Intercept) | 1·92 (1·02, 3·62) | 0·049 |
| Infection | 4·58 (3·33, 6·32) | <0·0001 |
| Diagnosis - Arthritis | 1·66 (1·06, 2·58) | 0·031 |
| Age (years) | 0·10 (0·99, 1·01) | 0·83 |
| Hypertension | 0·84 (0·47, 1·50) | 0·55 |
| Smoking | 0·80 (0·34, 1·85) | 0·60 |
| Combination therapy | 0·81 (0·57, 1·16) | 0·26 |
| Vaccine type - mRNA-1273 | 0·95 (0·51, 1·80) | 0·89 |
| Vaccine type - heterologous | 0·95 (0·69, 1·33) | 0·79 |
| Nucleocapsid Ab levels – three vaccine doses |  |  |
| (Intercept) | 1·77 (0·84, 3·73) | 0·14 |
| Infection | 4·61 (3·36, 6·35) | <0·0001 |
| Diagnosis - Arthritis | 0·85 (0·48, 1·52) | 0·58 |
| Age (years) | 1·00 (0·99, 1·01) | 0·86 |
| Hypertension | 0·92 (0·62, 1·37) | 0·67 |
| Smoking | 1·09 (0·62, 1·92) | 0·77 |
| Combination therapy | 1·00 (0·72, 1·39) | 0·99 |
| Vaccine type - mRNA-1273 | 0·83 (0·49, 1·41) | 0·49 |
| Vaccine type - heterologous | 1·02 (0·73, 1·42) | 0·90 |
| Percentage CD4 responses – three vaccine doses |  |  |
| (Intercept) | 0·076 (0·017, 0·252) | 0·00011 |
| Infection | 0·81 (0·39, 1·66) | 0·56 |
| Diagnosis - IBD | 0·79 (0·36, 1·75) | 0·56 |
| Diagnosis - Arthritis | 0·43 (0·12, 1·51) | 0·19 |
| Age (years) | 0·99 (0·97, 1·02) | 0·49 |
| Combination therapy | 1·24 (0·54, 2·82) | 0·62 |
| Vaccine type - mRNA-1273 | 2·08 (0·56, 7·67) | 0·27 |
| Vaccine type - heterologous | 2·33 (1·17, 4·67) | 0·018 |
| Percentage CD4 responses – four vaccine doses |  |  |
| (Intercept) | 0·177 (0·043, 0·721) | 0·019 |
| Infection | 1·53 (0·80, 2·92) | 0·20 |
| Diagnosis - Arthritis | 0·44 (0·14, 1·32) | 0·15 |
| Age (years) | 0·98 (0·96, 1·00) | 0·11 |
| Combination therapy | 0·76 (0·40, 1·46) | 0·41 |
| Vaccine type - mRNA-1273 | 1·52 (0·50, 4·65) | 0·46 |
| Vaccine type - heterologous | 1·38 (0·71, 2·68) | 0·35 |
| Percentage CD8 responses – three vaccine doses |  |  |
| (Intercept) | 0·004 (0·0009, 0·015) | <0·0001 |
| Infection | 1·63 (0·77, 3·45) | 0·21 |
| Diagnosis - IBD | 2·92 (1·27, 6·70) | 0·013 |
| Diagnosis - Arthritis | 0·15 (0·04, 0·52) | 0·0036 |
| Age (years) | 1·02 (0·10, 1·05) | 0·085 |
| Combination therapy | 0·40 (0·17, 0·95) | 0·039 |
| Vaccine type - mRNA-1273 | 1·60 (0·41, 6·21) | 0·50 |
| Vaccine type - heterologous | 1·54 (0·74, 3·17) | 0·25 |
| Percentage CD8 responses – four vaccine doses |  |  |
| (Intercept) | 0·0536 (0·005, 0·596) | 0·020 |
| Infection | 1·66 (0·55, 4·98) | 0·37 |
| Diagnosis - Arthritis | 0·30 (0·04, 2·04) | 0·22 |
| Age (years) | 0·99 (0·95, 1·03) | 0·65 |
| Combination therapy | 0·86 (0·28, 2·59) | 0·78 |
| Vaccine type - mRNA-1273 | 1·13 (0·16, 7·73) | 0·90 |
| Vaccine type - heterologous | 1·15 (0·37, 3·63) | 0·81 |
| CD4 polyfunctionality (PFS) – three vaccine doses† |  |  |
| (Intercept) | 0·242 (0·131, 0·353) | <0·0001 |
| Infection | -0·004 (-0·051, 0·042) | 0·86 |
| Diagnosis - IBD | 0·05 (-0·034, 0·134) | 0·25 |
| Diagnosis - Arthritis | -0·018 (-0·112, 0·076) | 0·71 |
| Age (years) | 0·001 (0, 0·002) | 0·19 |
| Combination therapy | -0·002 (-0·043, 0·038) | 0·91 |
| Vaccine type - mRNA-1273 | 0·107 (0·04, 0·175) | 0·0022 |
| Vaccine type - heterologous | 0·09 (0·048, 0·133) | <0·0001 |
| CD4 polyfunctionality – four vaccine doses† |  |  |
| (Intercept) | 0·45 (0·345, 0·555) | <0·0001 |
| Infection | -0·004 (-0·055, 0·047) | 0·89 |
| Diagnosis - Arthritis | -0·014 (-0·098, 0·071) | 0·75 |
| Age (years) | -0·001 (-0·003, 0) | 0·13 |
| Combination therapy | 0·003 (-0·048, 0·053) | 0·92 |
| Vaccine type - mRNA-1273 | 0·084 (-0·009, 0·177) | 0·081 |
| Vaccine type - heterologous | 0·037 (-0·013, 0·088) | 0·15 |
| CD8 polyfunctionality – three vaccine doses |  |  |
| (Intercept) | 0·086 (0·019, 0·385) | 0·0018 |
| Infection | 2·26 (1·20, 4·25) | 0·013 |
| Diagnosis - IBD | 1·49 (0·49, 4·60) | 0·49 |
| Diagnosis - Arthritis | 0·96 (0·27, 3·40) | 0·95 |
| Age (years) | 0·99 (0·97, 1·01) | 0·54 |
| Combination therapy | 0·69 (0·40, 1·19) | 0·19 |
| Vaccine type - mRNA-1273 | 0·85 (0·34, 2·10) | 0·72 |
| Vaccine type - heterologous | 1·15 (0·65, 2·05) | 0·63 |
| CD8 polyfunctionality – four vaccine doses |  |  |
| (Intercept) | 0·252 (0·077, 0·820) | 0·026 |
| Infection | 1·42 (0·79, 2·54) | 0·25 |
| Diagnosis - Arthritis | 0·63 (0·24, 1·62) | 0·34 |
| Age (years) | 0·99 (0·97, 1·01) | 0·26 |
| Combination therapy | 1·15 (0·65, 2·04) | 0·63 |
| Vaccine type - mRNA-1273 | 1·27 (0·45, 3·61) | 0·65 |
| Vaccine type - heterologous | 0·99 (0·56, 1·75) | 0·97 |

† Multiple regression analysis was carried out with log transformed data for all values except for CD4 PFS, which was carried out with non-log transformed data. Estimated effects for CD4 PFS are therefore additive.

# **Section 2. Supplementary figures**

## **Supplementary figure 1. Flow cytometry gating strategy**

For manual gating to determine the frequency of responding T cells, events were gated for time then for single cells, lymphocytes, live cells, CD3+ cells and CD4+ or CD8+ T cells. Activated CD4 T cells were gated as CD40L+ TNFα+ and activated CD8 T cells were gated as IFN-γ+ TNFα+. Background activation of unstimulated cells was subtracted from spike or non-spike stimulated cells for each sample.


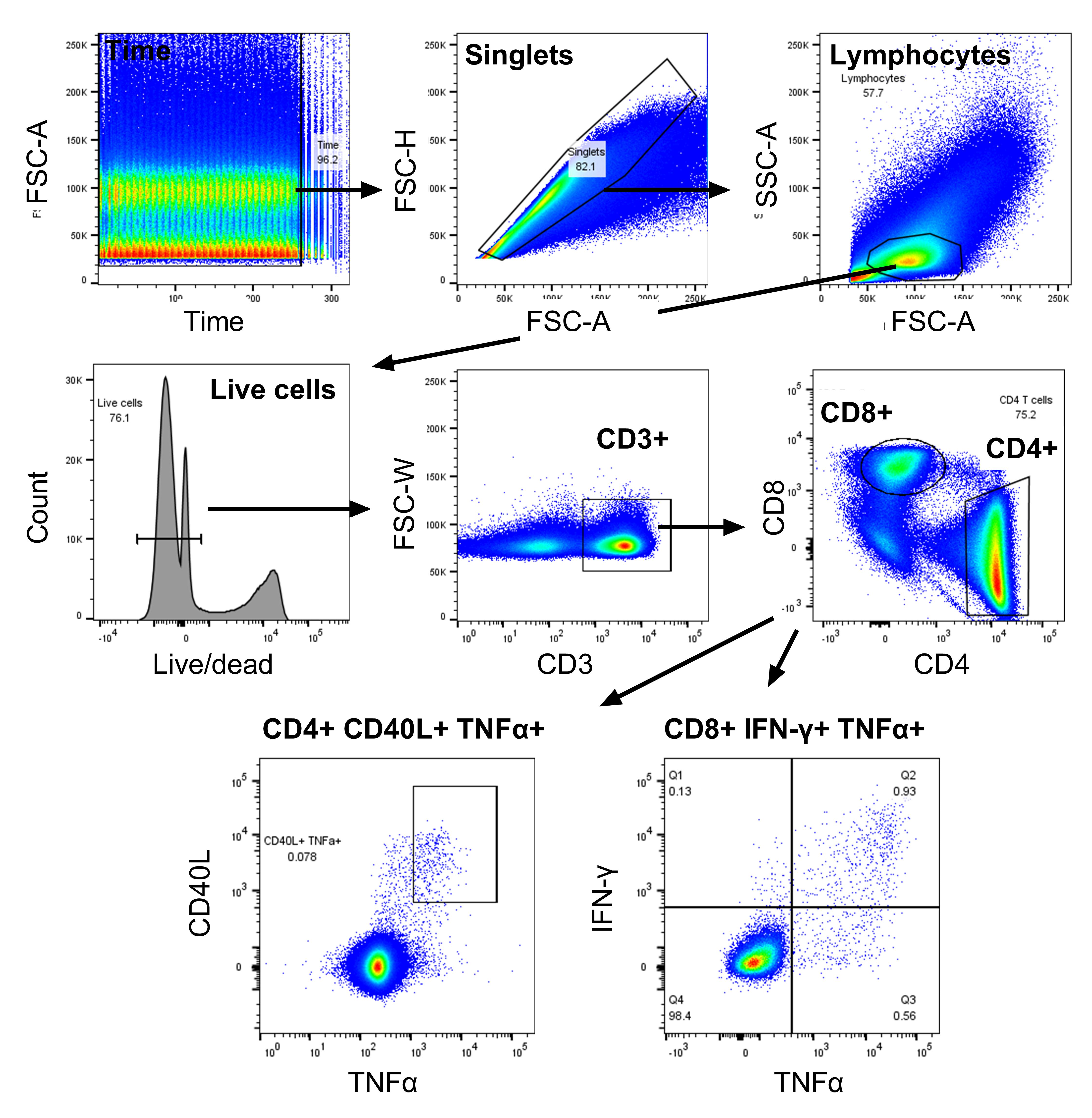


## **Supplementary figure 2. COMPASS heatmap showing mean posterior probabilities of spike-specific responses**

Columns correspond to different combinations of cytokines and activation markers (IL-2, IFN-γ, TNFα, CD40L and CD137) modelled by COMPASS ranging from expression of one marker only (light blue, leftmost columns) up to all five markers (pink, rightmost column). Each row is one individual. Donors are grouped by healthy control (HD), inflammatory bowel disease (IBD) and arthritis (RA). Vaccine time point is indicated on the righthand side (v0, unvaccinated; v2, second vaccine dose; etc). Each cell of the heatmap shows the probability that the corresponding cell-subset (column) exhibits an antigen-specific response in the corresponding subject (row), where the probability is color-coded from white (zero) to purple (one). Polyfunctionality is considered the expression of two or more markers.

1. CD4 T cell posterior probabilities

1. CD8 T cell posterior probabilities

## **Supplementary figure 3. Directed acyclic graph for study design**

The directed acyclic graph (DAG) for this study. Exposure (yellow box) and outcome (blue box) are defined as TNFi treatment and immune response after vaccination respectively. Grey boxes indicate adjusted variables, white circles indicate unobserved variables, and red boxes indicate ancestors of exposure and outcome. The green line linking exposure and outcome shows the causal path. The minimal sufficient adjustment set contains age, arthritis, hypertension, IBD, smoking, and vaccine type for estimating the total effect of TNFi treatment on immune responses after vaccination. This figure was made using the web package DAGitty v.3.1.


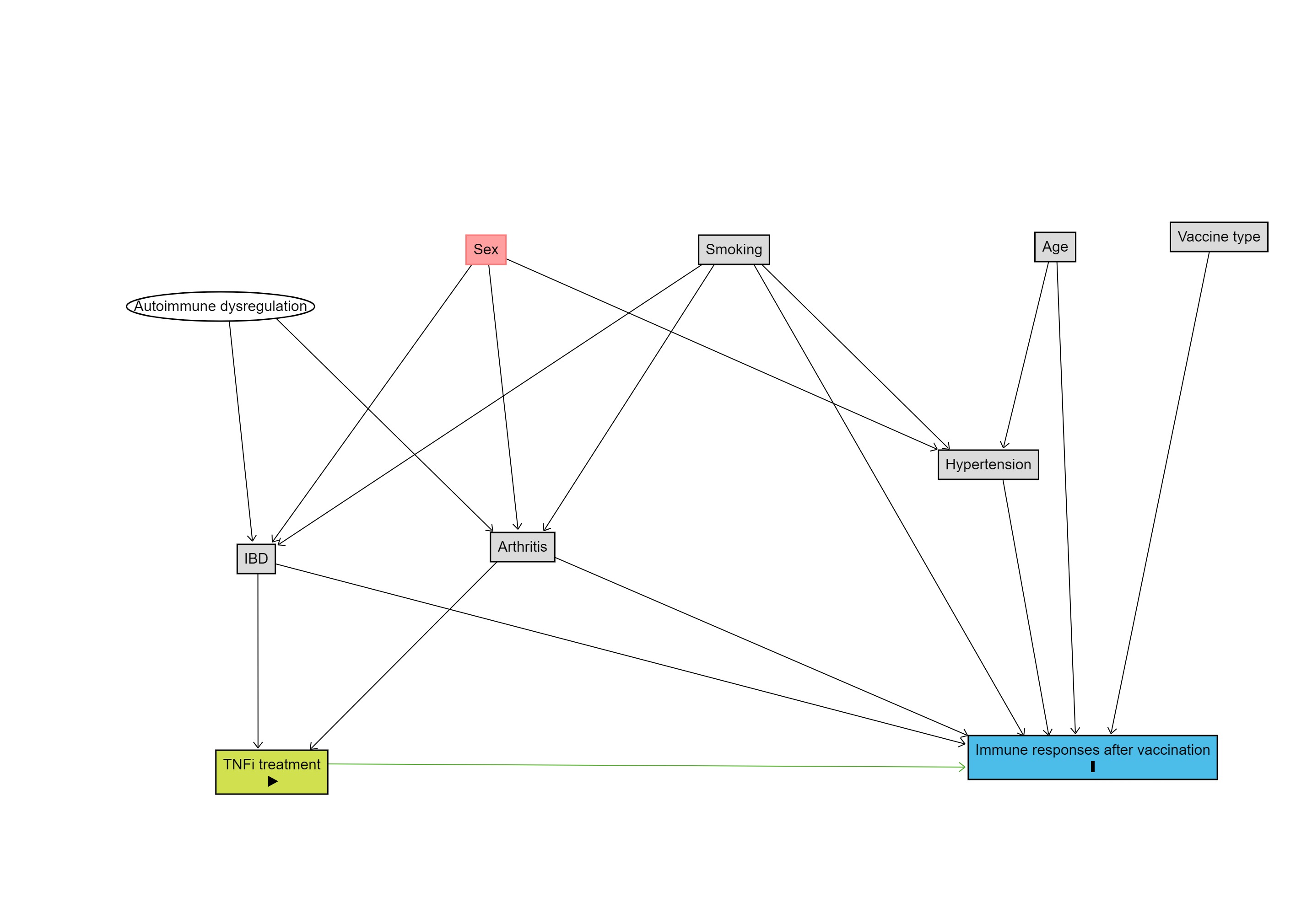


## **Supplementary figure 4. Associations between T cell responses and humoral responses**

CD4 and CD8 T cell responses in all patients on TNFi were positively associated after three vaccine doses (a). CD4 (red) and CD8 (blue) T cell responses also showed a positive association with antibody levels (BAU/ml, anti-RBD for Wuhan-Hu-1 strain) after three vaccine doses (b). Graphs were plotted using local polynomial regression fitting (loess) and shaded areas indicate the 95% confidence intervals.


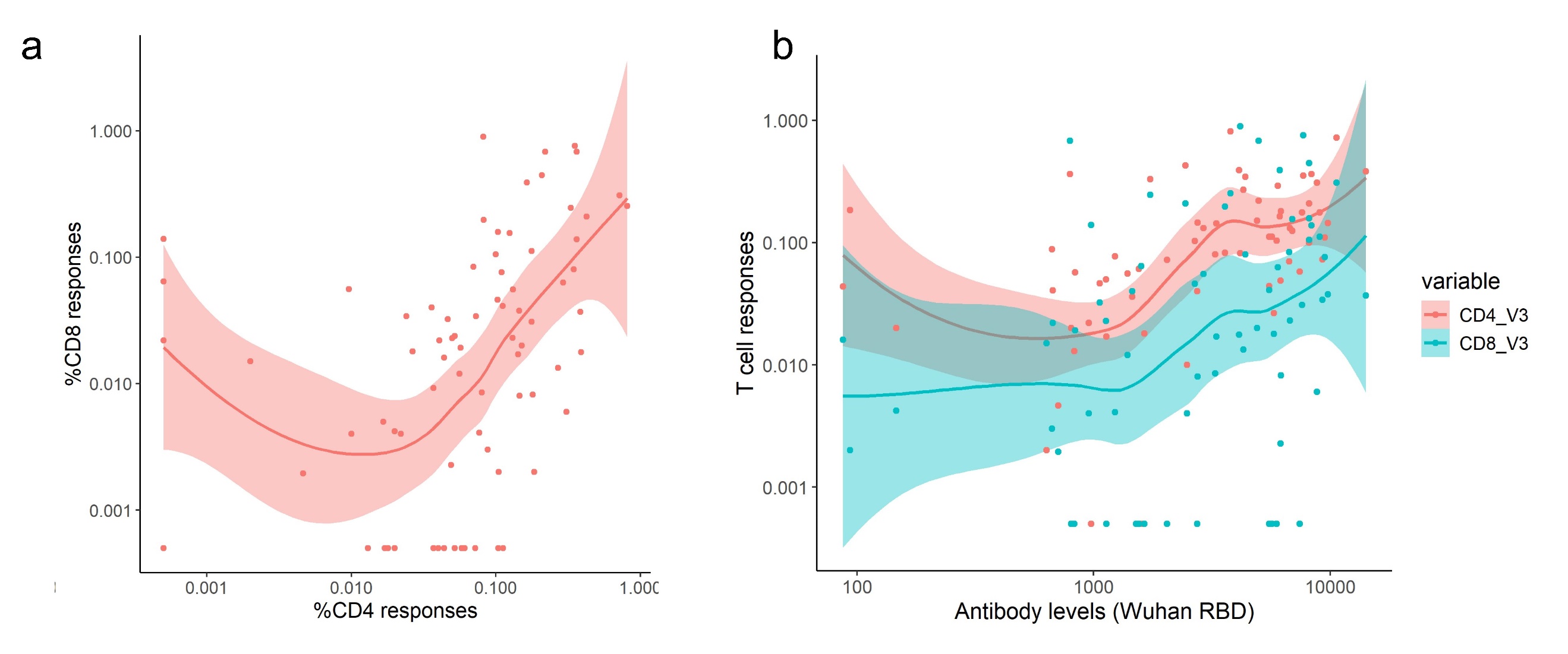


## **Supplementary figure 5. Effect of TNFi treatment on vaccine responses**

All patients with IBD were split by treatment group: adalimumab (green) or infliximab (purple) monotherapy, or in combination with azathioprine or methotrexate (darker shades). Responses were plotted after each vaccination time point (V2, V3, and V4). No significant differences between either adalimumab and infliximab monotherapy or the relevant monotherapy in combination with a secondary treatment were observed. Patients treated with golimumab (n=2 for monotherapy, n=1 for golimumab + methotrexate) were excluded from this comparison due to insufficient numbers for statistical comparison. Box-and-whisker plots indicate median, IQR and 1.5×IQR from Q1 and Q3. Statistical analysis was performed by Wilcoxon rank-sum test with FDR correction; adjusted p-values shown for all comparisons. The table indicates treatment combinations and numbers per group. All comparisons were non-significant.


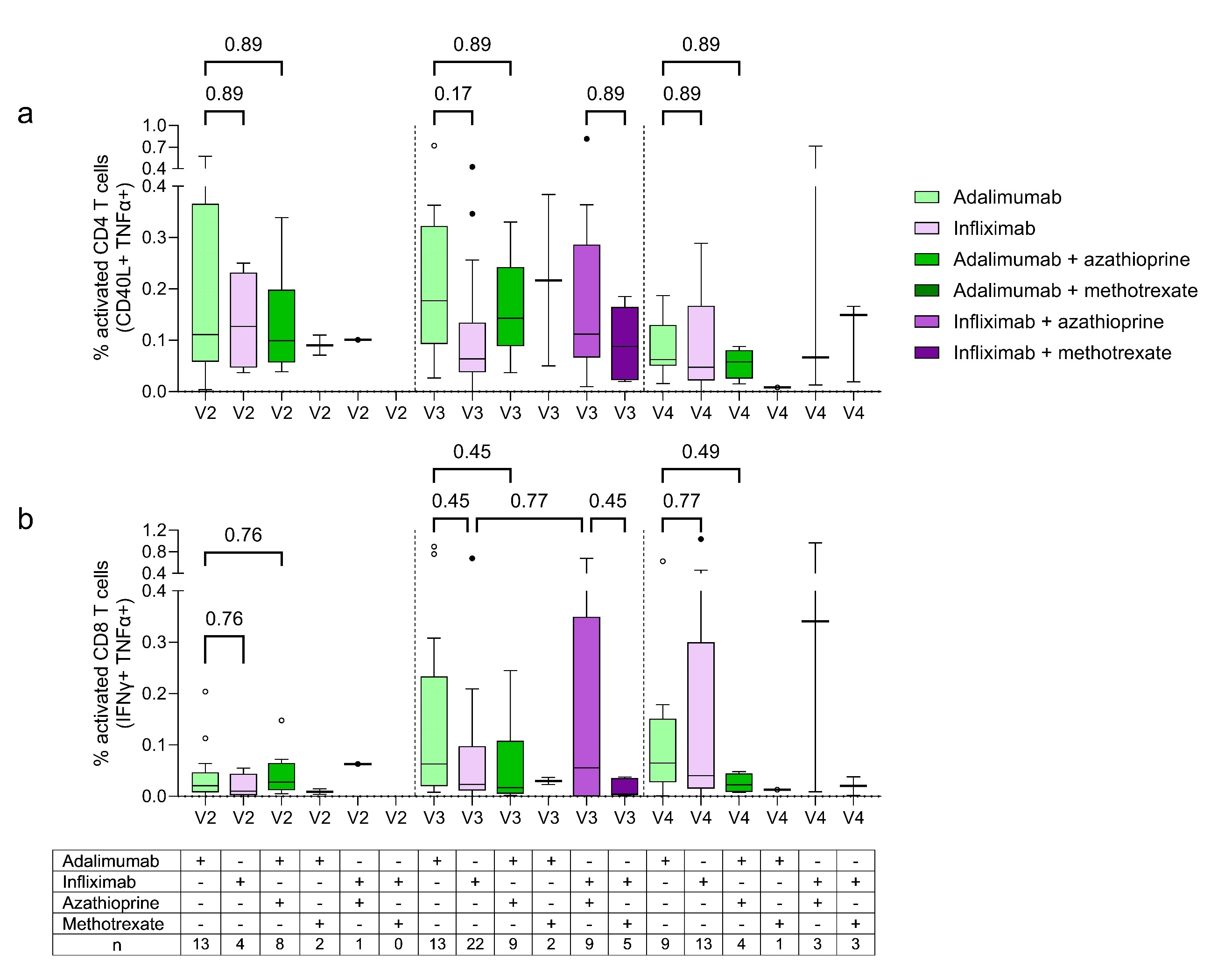


## **Supplementary figure 6. Non-spike responses after breakthrough infection by diagnosis group**

After a breakthrough infection (BTI), the majority of patients made CD4 and CD8 T cell responses to SARS-CoV-2 nucleocapsid and membrane peptides. A comparison of patients with IBD (red) vs. arthritis (blue) did not show significant differences in their post-BTI CD4 (a) or CD8 (b) T cell responses, although this analysis is limited by low numbers of patients with arthritis (n=5 for nucleocapsid and membrane responses). Statistical analysis was performed by Wilcoxon rank-sum test with FDR correction; adjusted p-values shown for all comparisons. Numbers per group are indicated below each bar. Box-and-whisker plots indicate median, IQR and 1.5×IQR from Q1 and Q3. Pale shades indicate the pre-BTI response after three vaccine doses; darker shades indicate the post-BTI response after three or four vaccine doses. Pre-BTI responses from nucleocapsid and membrane are not shown.


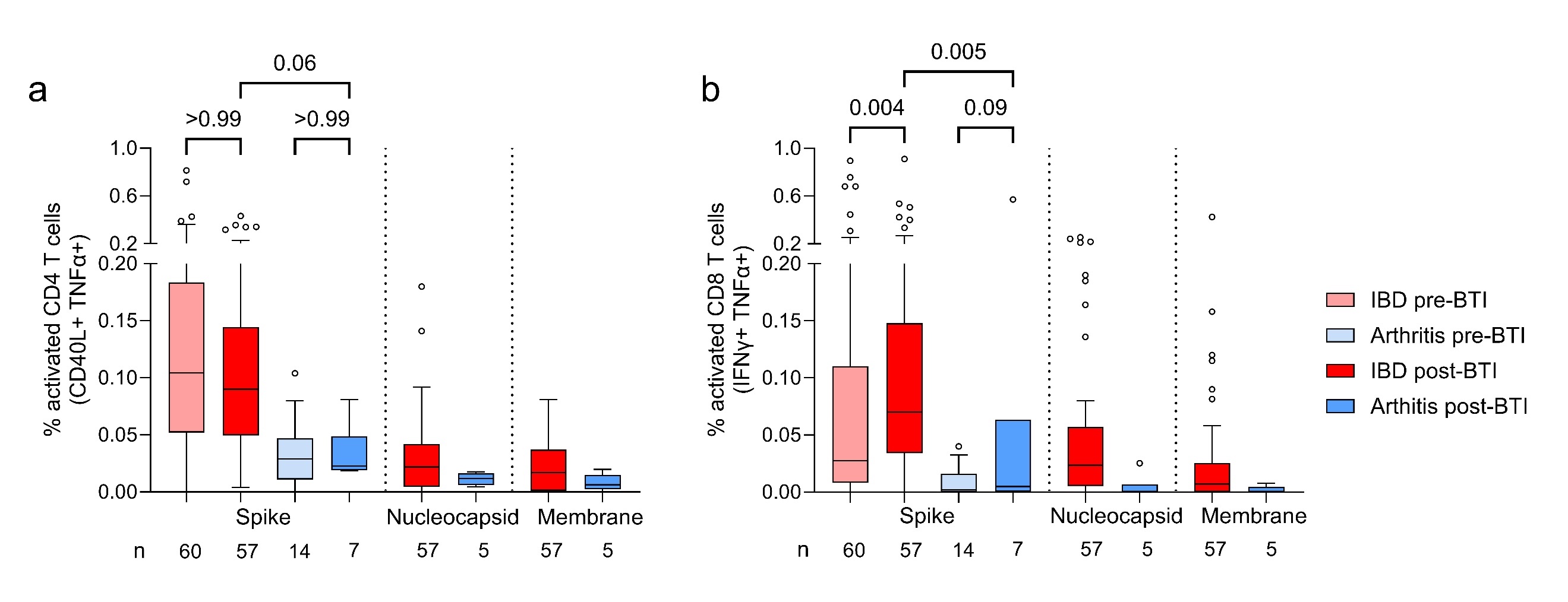


## **Supplementary figure 7. Nucleocapsid and membrane responses are not associated with spike-specific responses after breakthrough infections**

After a breakthrough infection, patients on TNFi made CD4 and CD8 responses to SARS-CoV-2 nucleocapsid (ncap, red) and membrane (blue) peptides. These non-spike responses did not show strong associations with spike-specific responses. Graphs were plotted using local polynomial regression fitting (loess). Shaded areas indicate 95% confidence intervals.


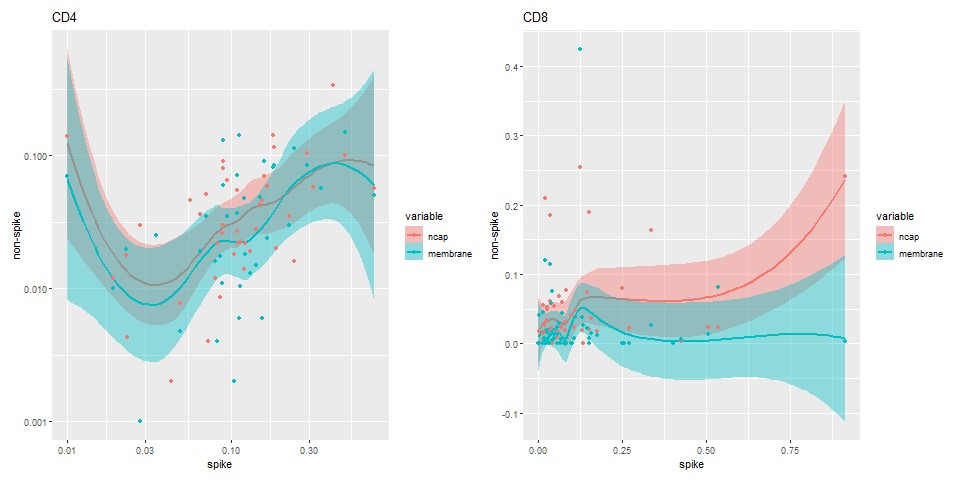

Supplement: Appendix [file mmc1.docx]
